# Progress in Genito-Urinary Surgery

**Published:** 1902-09-20

**Authors:** 


### Progress in Genito-Urinary Surgery.

**The Operative Treatment of Enlarged Prostate.**—J. W. T. Walker[1] has given an interesting *resumé* of a number of recent papers on this much discussed subject, from which we abstract the following remarks. The genital operations, castration and vasectomy, have now had an extensive trial. The most recent statistics seem to be those of Wood, of Philadelphia, who collected 130 well reported cases. Of these 90 per cent. were benefited. In 57 per cent. the function of micturition was improved, and in 18·5 per cent. the residual urine diminished in quantity or disappeared. The mortality was 8 per cent., and in 4 per cent. mental disturbance followed but was recovered from. With regard to mental effect castration and vasectomy are about on the same footing. The latter operation, as shown in an analysis of 193 cases, produced similar effects but only in a much smaller percentage of cases, and its mortality was 6·7 per cent. H. Fenwick, discussing the effects of these operations on the prostate states that the gland is not dwarfed as felt by the rectum, but becomes broader and flatter, and if both sides are affected the sulcus or interlobar fissure is replaced by a deep groove. The lobes also become firmer and less elastic. The deep sulcus denotes atrophy of the parts of the prostate adjacent to the urethra and the relief of symptoms is due to the atrophy of this part of the gland. The author believes that if the lobes of the enlarged prostate are flat and feel fibrous, and if the sulcus is broad and deep, then the case is one in which castration will not be of much use. Castration is of infinitely greater value than vasectomy. Two years ago Guiteras stated that in the United States castration had been abandoned in the treatment of enlarged prostate. Prostotomy by Bottini's method of galvano-caustic incision of the enlarged gland, though little favoured in this country, has been much practised in Germany and America. Willy Meyer advises that the operation should be performed as soon as it is necessary to resort to self-catheterisation. Horwitz is a strong advocate of the operation. His results in 36 cases were such that in only three was there a tendency to recurrence of obstructive symptoms, and in these a repetition of the operation under local anæsthesia gave satisfactory results. He records one case in which he had an opportunity of viewing the urethral orifice through a supra-pubic opening seven months after the Bottini operation. He found that all obstruction had been removed and that a deep furrow existed in the middle line of the median lobe. Parker Syms takes a very different view of the operation. He believes it to be an absolutely unsurgical and unscientific procedure, and that it is dangerous and incomplete. Other results of the Bottini operation may be gathered from the statistics of Horwitz, who collected 888 cases from all sources. The mortality was 5·7 per cent., in 10 per cent. no benefit was derived, and 84 per cent. were cured or improved. Bolton Bangs records 36 cases with the results that in 60 per cent. no catheter was required after the operation; in 20 per cent. the catheter was "very seldom" required; and in 20 per cent. there was no improvement or very little. Prostatectomy may be performed by the perineal route or supra-pubically. Guiteras recommends the perineal method when the enlargement is principally of the lateral lobes. According to Fenwick there are two essential varieties of enlargement to be differentiated, viz., the small, tough, flat *unshellable* prostate, which generally has a filled-up sulcus, and the large, soft, elastic, projecting *shellable* prostate, which has a fine line of sulcus. The majority of French surgeons follow Nicoll, of Glasgow, and attack the prostate from the perineum. Freyer has published a series of 8 cases in which he removed the entire enlarged prostate in its capsule, leaving the prostatic urethra intact. Seven of the cases were completely successful in all respects. One case died of acute mania on the twenty-second day after the operation.

**Imperfectly Descended Testis.**—W. McAdam Eccles[2] very fully considered the physiology and pathology of the imperfectly descended testis in the Hunterian Lectures of this year. The following are some of the considerations he brought forward. There are two causes of ectopia or misplaced testicles, viz., abnormal traction exerted by the gubernaculum testis, and displacement by an advancing hernia. When the testicle is found in Scarpa's triangle the spermatic cord can nearly always be traced, not through the femoral ring, but up to and through the superficial abdominal ring, and it is very doubtful if the testicle has ever passed into this position by descending through the crural canal. About 5 per cent. of imperfectly descended testicles are of normal size, and 95 per cent. smaller than natural. Such testicles have very definite differences in structure as compared with the normal. In them the tubules are fewer in number, smaller, and more widely separated from one another by interstitial tissue than in normal testicles. In some there are more interstitial stroma cells present than usual. Possibly these interstitial cells are concerned in the production of an internal secretion which is important for the development of virile characteristics at puberty. Double arrest of the testicles with imperfect development as a rule implies sterility on the part of the man, though he may be perfectly virile. There is real doubt whether a testicle which was arrested at birth but was brought down in early life into the scrotum, ever so developed as to generate spermatozoa, though this has been stated to occur. When one testicle is arrested the other is very rarely larger than natural. A single well-developed testicle is sufficient to provide all that is needed for the proper growth of the individual. Where the cæcum has failed to reach the iliac-fossa the right testicle has not usually passed out of the abdomen. Undescended testicle is usually met with in cases of hermaphroditism both in the true forms in which one ovary or more and one testicle or more exist, and in the false varieties in which there is an external appearance of the organs of generation of one sex with the internal organs proper to the other sex, and in the mixed cases. If there are bodies in the inguinal canals, and some doubt as to the sex as shown by the external organs, the person should be looked upon as a male rather than as a female. The undescended or ectopic testicle is very prone to attacks of inflammation excited by mechanical means. Acute hydroceles are often associated with the orchitis, and the fluid which collects may fill the lower part of the sac and obscure the testicle. Atrophy of the testicle is a frequent result of the inflammation. Sometimes the testicle remains considerably enlarged and may then simulate a new growth. In a few instances peritonitis has resulted from the inflammation of the testicle, whether traumatic or gonorrhœal. Nearly 50 per cent. of the recorded cases of torsion of the spermatic cord are in connection with arrested testes. Malignant growth does not appear to affect the undescended testicle any more frequently than the fully descended one.

**Treatment of Hydrocele.**—William B. Coley [3] has written on the treatment of this affection by the carbolic acid method. Levis, who introduced the treatment in 1881, had used from 30 grains to a drachm and a half of the acid, without any poisoning effects, and the patients had not been incapacitated from attending to their usual work. Nearly all the hydroceles of infancy either disappeared spontaneously or could be cured by the application of equal parts of tincture of iodine and tincture of belladonna. For the adult the writer now used small injections of carbolic acid. Each injection consisted of two and a half minims of Schering's carbolic acid, with the smallest quantity of glycerine necessary to liquefy the acid. The results had been just as good as from the larger injections, and were without the risk and discomfort of those. It was very important to have the carbolic acid solution sufficiently concentrated. Ordinary commercial carbolic acid had not given nearly such good results as the more concentrated acid of Schering. The author used a small double trochar for the injections. The hydrocele fluid was very thoroughly evacuated through the outer trochar, and then the carbolic injected through the inner trochar, which was attached to a hypodermic syringe. With this there was no trouble from leakage of the acid, and it was a most useful instrument for enabling the operator to squeeze out every drop of the hydrocele fluid and to make sure that the carbolic acid was then injected into the sac. The author believes that the carbolic-acid injection method is the best yet devised for the cure of hydrocele. He had treated 36 cases with the small injections with success.

[1] Practitioner, May 1902, p. 576. [2] Lancet, March 1st, 1902, p. 569, and March 15, p. 722. [3] Med. Rec., Feb. 8, 1902, p. 235.

